# Massive encapsulation of larval Anguillicoloides crassus in the intestinal wall of Japanese eels

**DOI:** 10.1186/1756-3305-2-48

**Published:** 2009-10-15

**Authors:** Emanuel G Heitlinger, Dominik R Laetsch, Urszula Weclawski, Yu-San Han, Horst Taraschewski

**Affiliations:** 1 Department of Ecology and Parasitology, Zoological Institute 1, University of Karlsruhe, Kornblumenstrasse 13, Karlsruhe, Germany; 2 Institute of Evolutionary Biology, The Ashworth laboratories, The University of Edinburgh, King's Buildings Campus, Edinburgh, UK; 3 Institute of Fisheries Science, College of Life Science, National Taiwan University, Taipei, Taiwan

## Abstract

**Background:**

Within the last 25 years, after the introduction of the swimbladder nematode *Anguillicoloides *crassus from East-Asia to Europe, a body of work has aggregated on the host parasite interactions in the acquired host *Anguilla anguilla*. Despite the emerging evolutionary interest there is still a lack of knowledge about host parasite relations of *A. crassus *in its natural host *Anguilla japonica*. We examined the *Anguillicoloides *infections of wild-caught Japanese eels as well as from aquacultured specimens in Taiwan with respect to the fate of migratory L3 larvae and performed infection experiments with Japanese eels.

**Results:**

Inside the intestinal wall of cultured eels, where the infective pressure was higher than among wild eels, we found large numbers of granuloma-like cysts. In a few eels these cysts contained nematodes still recognizable as L3 larvae of *A. crassus*, while in most cases the content of these capsules was degraded to amorphous matter. Occurrence of these objects was correlated with the number of encapsulated larvae in the swimbladder wall. We were able to show, that the cysts contained disintegrated L3 larvae by amplification and subsequent sequencing of large subunit ribosomal rRNA. Furthermore we identified repeated infections with high doses of larvae as prerequisites for the processes of encapsulation in infection experiments.

**Conclusion:**

Under high infective pressure a large percentage of L3 larvae of *A. crassus *coming from the gut lumen are eliminated by the natural host within its intestinal tissue. It is possible to reproduce this condition in infection experiments. We provide a fast, easy and reliable PCR-based method for identification of encapsulated swimbladder parasites.

## Background

*Anguillicoloides crassus *(formerly known as *Anguillicola crassus*) Kuwahara, Niimi et Itagaki, 1974 [1,2] is a swimbladder nematode naturally parasitizing the Japanese eel (*Anguilla japonica*) indigenous to East-Asia. After a single introduction [3] to Germany in the early 1980s *A. crassus *has colonized almost all populations of the European eel (*Anguilla anguilla*) [4]. Since the 1990s populations of the American eel (*Anguilla rostrata*) have been colonized as novel hosts [5-7] and finally it has been detected in three indigenous *Anguilla *species on the island of Reunion near Madagascar [8].

In Asia, as well as in the introduced ranges, copepods and ostracods serve as intermediate hosts of *A. crassus *[9], in which L2 larvae develop to L3 larvae, infective to the final host. Once ingested by an eel they migrate through the intestinal wall and via the body cavity into the swimbladder wall [10], i.a. using a trypsin-like proteinase [11]. In the swimbladder wall L3 larvae hatch to L4 larvae. After a final moult from L4 to preadult the parasites inhabit the lumen of the swimbladder, where they eventually mate. Eggs containing L2 larvae are released via the ductus pneumaticus into the eels gut and finally into the water [12].

Within the novel range and hosts, conspicuously elevated prevalences and intensities of infection occur (reviewed in [4] and [13]). These differences in abundance of *A. crassus *in East Asia compared to Europe are commonly attributed to the different host-parasite relations in the final eel host permitting a differential survival of the larval and the adult parasites [14]. Recently, data from experimental infections of European eels with *A. crassus *have been published [15]. They show that the parasite undergoes (under experimental conditions) a density-dependent regulation keeping the number of worms within a certain range.

Despite this scientific interest in this nematode and the huge amount of data from infections of the European eel, there is still a lack of knowledge about the interaction of the natural host *A. japonica *with this parasite in East Asia.

Comparative studies on Japanese eels under different conditions (i.e. free living versus cultured) are rare:

However, one study demonstrated a higher abundance of *A. crassus *in Japanese eels from an aquaculture compared to a a river [16]. Accordingly, the recruitment of L3 larvae in farmed eels was much higher than in wild conspecifics. In the present study we investigated the fate of L3 larvae in Japanese eels under different infection pressures.

Eels were caught in the southwest of Taiwan in the Kao-Ping-river and in an adjacent aquaculture. In addition to the swimbladder (wall and lumen), we examined the intestinal wall, the first host-tissue barrier to larval *A. crassus *(L3) during their migration towards the swimbladder. Inside the intestinal wall many granuloma-like capsules could be detected partly harboring nematode larvae, which were still alive. In others it was hard to decide whether the objects contained were helminths, fungi or some other material because their content was only visible as amorphous matter. It was therefore an aim of this study to identify the enclosed objects. This was pursued by amplification and subsequent sequencing of a species specific marker gene (LSU rDNA) from these capsules. Furthermore the process of encapsulation was demonstrated under experimental conditions.

## Results

### Eel data

In total 191 Japanese eels were examined for this study, 40 from Kaoping River and 151 from an adjacent aquaculture in SW Taiwan. Cultured eels were significantly (p < 0.001) larger (53.0 cm ± 56.78; mean ± SD) than wild eels (40.3 cm ± 95.73; mean ± SD), accordingly the weight of cultured eels was significantly (p < 0.001) higher (169.8 g ± 78.78; mean ± SD) than that of wild eels (93.0 g ± 98.64; mean ± SD). The sex ratio was skewed towards females, strongly in wild eels only slightly in cultured eels. The majority of dissected eels were immature juveniles (yellow stage); only 17 cultured and 1 wild eel were mature silver eels. Hardly any helminth parasites other than *A. crassus *were found in the swimbladder or intestine of the investigated eels: an intestinal cestode was found in one eel from Kaoping river and a small intestinal trematode in two eels, both from aquaculture.

### Epidemiological parameters

**Figure 1:**
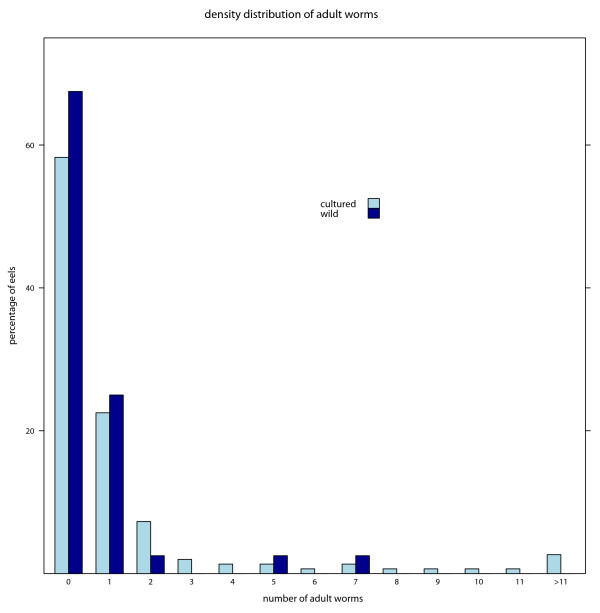
**Frequency distribution of A. crassus in wild and cultured Japanese eels**. Observed frequency distribution of (a) adult and (b) larval *A. crassus *from wild (n= 151) and cultured (n = 40) eels.

From the epidemiological parameters (table 1) a clear pattern emerges: prevalences, mean intensities and abundances are lower in the investigated wild eels than in eels from aquaculture. These differences are significant or close to significance if values are calculated for the total number of adults and larvae. If parameters are analyzed separately for larvae and adults the differences for larval parameters are highly significant, with higher prevalence, mean intensity and abundance in the cultured eels. The differences in adult parameters lose significance if analyzed under exclusion of larvae. The highest intensity for wild eels was 7 adults the maximal observed intensity in eels from aquaculture was 21 adults in one swimbladder. The frequency distribution of adult *A. crassus *per swimbladder approaches a negative binomial distribution (Figure 1). Compared to wild eels, a higher degree of aggregation is indicated in cultured eels compared to their wild conspecifics. Furthermore the degree of over-dispersion is elevated for larvae compared to adult parasites.

**Table 1:** Epidemiological parameters.

	**n**	**prevalence (%)**			**mean abundance**			**mean intensity**		
				
**source**		**adult**	**larval**	**total**	**adult**	**larval**	**total**	**adult**	**larval**	**total**
										
wild	40	32.5	2.5	32.5	0.6	0.05	0.65	1.85	2.0	2.0
± SD					± 1.37	± 0.32	± 1.42	± 1.91	± NA	± 1.91
										
cultured	151	41.7	25.82	47.7	1.4	1.0	2.4	3.48	3.85	5.01
± SD					± 3.32	± 3.0	± 5.0	± 4.48	± 4.88	± 6.26
										
comparison p		0.38	0.003	0.12	0.17	0.001	0.027	0.15	NA	0.038

A comparison of infections of wild and cultured populations of *Anguilla japonica *with *Anguillicoloides crassus*. Prevalences were compared using a chi-square test and abundances and intensities with Wilcoxon rank sum tests. NA indicates not available.

Finally the reproductive success of parasites seems to be almost equal: Despite the elevated levels of larvae (and to a lesser extent adults) the proportion of hosts in which reproduction was successful is almost equal: In 2 (5%) of the wild eels L2 larvae were observed and in 9 (6.1%) cultured eels L2 larvae were present in the swimbladder.

### Finding of dead larvae

**Figure 2:**
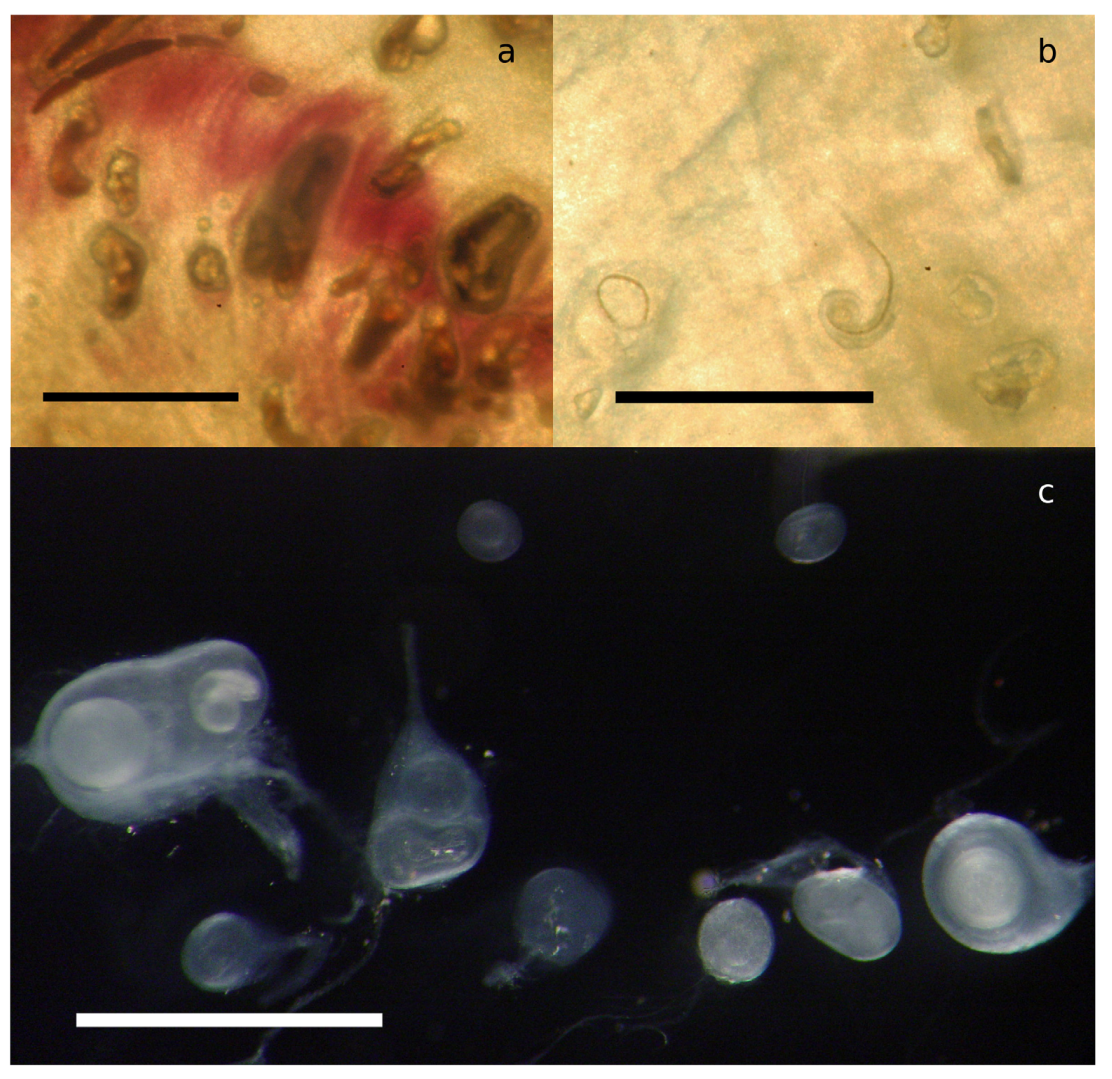
**Encapsulated larvae of A. crassus in the intestinal and swimbladder-wall of Japanese eels**. Encapsulated larvae of *A. crassus *from the swimbladder wall (a) and the intestinal wall (b and c). Pictures a and b are taken from unfixed tissue during dissection using transmitted light under a stereo microscope. Picture c is taken during preparation of intestinal capsules for PCR from fixed tissue using incident light. Scales indicate 1 mm.

Cysts ranging from capsules of amorphous content to capsules containing larvae of *A. crassus *(table 2, figure 2) still recognizable as such by visual inspection were counted in the wall of the swimbladder. Data on these swimbladder capsules was collected from 141 of the cultured eels and 35 of the wild eels. These capsules often concentrated around the rete mirabili, have been assumed to be dead, encapsulated larvae by other authors [16].

**Table 2:** Parameters describing the encapsulation of A. crassus larvae. A comparison of the extent of encapsulation of *A. crassus *in wild and cultured populations of *Anguilla japonica*. Comparisons carried out as described for table 1. Sb. caps. stands for capsules found in the swimbladder int. caps. for capsules in the intestinal wall. Sample size: n = 35 (wild) and n = 142 (cultured) for swimbladder capsules, n = 25 (wild) and n = 122 (cultured) for intestinal capsules.

	**prevalence (%)**		**mean abundance**		**mean intensity**	
		
**source**	**sb. caps.**	**int. caps.**	**sb. caps.**	**int. caps.**	**sb. caps.**	**int. caps.**
						
wild	14.29	3.45	0.51	0.03	3.6	1.0
± SD			± 1.74	± 0.19	± 3.44	± NA
						
cultured	74.65	44.27	34.57	346.01	46.31	781.64
± SD			± 62.33	± 942.74	± 68.32	± 1296.26
						
comparison p	<< 0.001	<< 0.001	<< 0.001	<< 0.001	0.008	NA

In one particularly interesting case hundreds of capsules were also found in fibrous tissue lumps surrounding the intestine and swimbladder inside the body cavity.

Additionally we observed large numbers (max. 4700) of comparable cysts in the intestinal wall of cultured eels (figure 2b), a finding not reported for *A. japonica *before. From the third day of dissactions we quantified these intestinal capsules leading to a sample of 122 cultured eels and 25 wild eels. In only one of the wild eels we found one capsule in the intestinal wall so our further investigation on this data concentrates on cultured eels. Higher numbers of these capsules were observed in the posterior regions than in the more anterior region of intestines.

**Figure 3:**
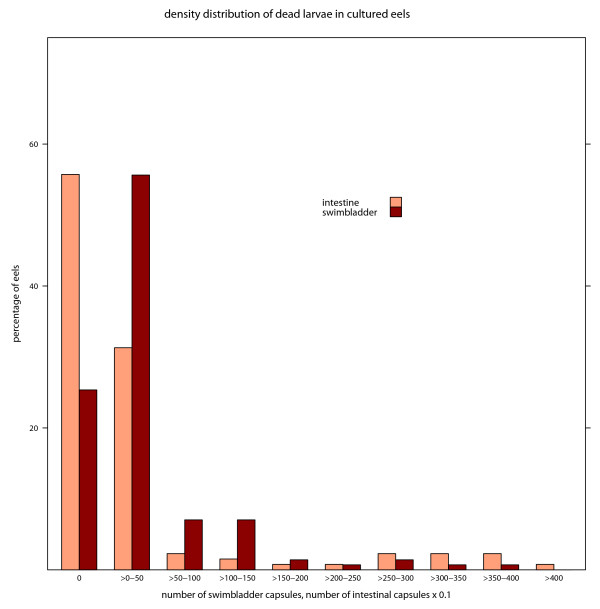
**Frequency distribution of dead A. crassus in the intestinal and swimbladder-wall of Japanese eels**. Observed frequency distribution of dead, encapsulated *A. crassus *in the wall of the swimbladder (n = 141) and the intestine (n = 122), note that the number of intestinal capsules has been transformed by a factor of 0.1 to fit the values for swimbladder capsules.

The frequency distribution of both capsules from the swimbladder and intestinal wall approaches a negative binomial distribution in cultured eels (figure 3). The same is true for swimbladder capsules of wild eels (data not shown). The degree of over-dispersion is more pronounced for intestinal capsules than for swimbladder capsules.

**Figure 4:**
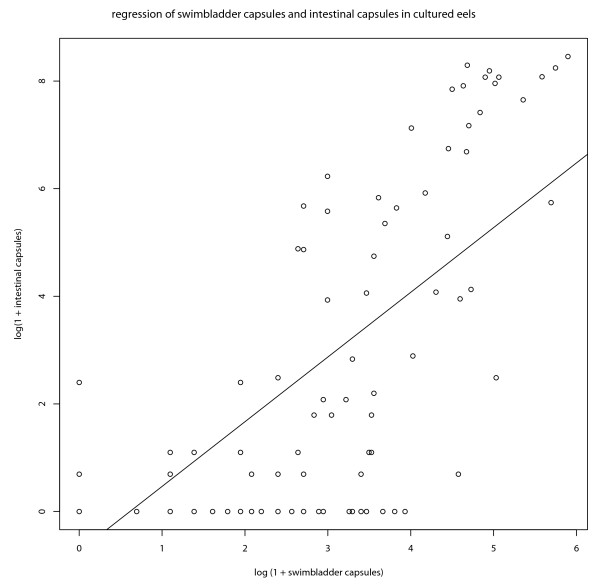
**Regression of encapsulated A. crassus in the intestinal wall vs. capsules from the swimbladder wall**. The log(1+x) transformed number of intestinal capsules from cultured eels as a function of the log(1+x) transformed number of swimbladder capsules. Formula for regression: *x *= -0.8335 + 1.2465*y*. The regression is highly significant (p < 0.001) Adjusted r^2 ^= 0.6021.

The simplest regression model using untransformed data predicts 11.55 intestinal capsules for each swimbladder capsule. In all regression models examined this regression of intestinal capsules on capsules in the swimbladder wall remained highly significant (p < 0.001), explaining 60% of the observed variation (r^2 ^> 0.621). This is also true for the simple regression model when both variables are log transformed (figure 4). High numbers of intestinal capsules are never observed in eels without capsules in their swimbladder wall.

In all appropriate generalized linear models (link function = log; quasipoisson), the number of swimbladder capsules remained the most significant explanatory variable for the number of intestinal capsules, followed by the number of (living) L3 larvae and L4 larvae.

### Amplification and sequencing of LSU rDNA from intestinal capsules

**Figure 5:**
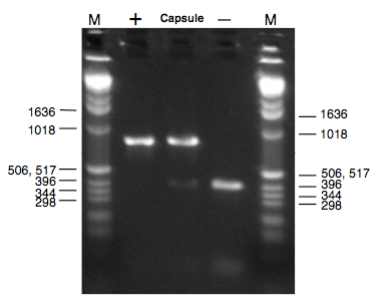
**Agarose gel showing two characteristic bands for amplification products from intestinal capsules**. Lanes are labeled as follows: "M" indicates the size-marker, "+" indicates the positive control (amplification product of a whole worm), "Capsule" a typical amplification product of an intestinal capsule and "-" the negative control (amplification product of uninfected intestinal tissue). Numbers correspond to the size of bands of the ladder (in bp).

PCR products analyzed on an agarose gel showed two clearly distinguishable bands at 300 bp and 800 bp. From 25 products of capsules whose shell had been manually destroyed before lysis, 15 showed a band at 800 bp and all 25 showed a second band at 300 bp. The 10 capsules where no 800 bp band was visible were among the smallest used for amplification. All 5 PCR products from capsules not manually opened showed only a band at 300 bp. Only this band at 300 bp was visible in all 10 negative control reactions carried out with intestinal tissue showing no signs of infection. Sequences obtained from 14 different bands at 800 bp showed 100% BLAST identity with the reference sequence generated from *A. crassus *(for accession and gi numbers see table 3). Identity to reference sequences from other *Anguillicoloides *sp. was 97% to *Anguillicoloides australiensis *97% to *Anguillicoloides novaezelandiae*, 96% to *Anguillicoloides papernai *and 96% to *Anguillicola globiceps*. The sequences obtained from bands at 300 bp showed a 94% BLAST identity with the LSU sequence for the American eel *Anguilla rostrata *available on GeneBank (GI: 1144501), although it shows poor coverage (84%). It is likely to be a chimeric sequence due to PCR recombination and biased amplification of the host large subunit ribosomal rDNA gene. This shows that the band at 800 bp clearly correspond to the LSU gene of *A. crassus *(see figure 5).

**Table 3:** Accession and gi numbers of generated sequences

**accession no.**	**gi no.**	**sequence name**	**from**
			
FJ748532	224814619	A. crassus isolate DL_CAPS_2A	intestinal Capsule: Taiwan
FJ748533	224814620	A. crassus isolate DL_CAPS_2C	intestinal Capsule: Taiwan
FJ748534	224814621	A. crassus isolate DL_CAPS_2D	intestinal Capsule: Taiwan
FJ748535	224814622	A. crassus isolate DL_CAPS_3A	intestinal Capsule: Taiwan
FJ748536	224814623	A. crassus isolate DL_CAPS_3C	intestinal Capsule: Taiwan
FJ748537	224814624	A. crassus isolate DL_CAPS_4A	intestinal Capsule: Taiwan
FJ748538	224814625	A. crassus isolate DL_CAPS_4B	intestinal Capsule: Taiwan
FJ748539	224814626	A. crassus isolate DL_CAPS_4C	intestinal Capsule: Taiwan
FJ748540	224814627	A. crassus isolate DL_CAPS_4D	intestinal Capsule: Taiwan
FJ748541	224814628	A. crassus isolate DL_CAPS_4E	intestinal Capsule: Taiwan
FJ748542	224814629	A. crassus isolate DL_CAPS_4F	intestinal Capsule: Taiwan
FJ748543	224814630	A. crassus isolate DL_CAPS_4G	intestinal Capsule: Taiwan
FJ748544	224814631	A. crassus isolate DL_CAPS_4H	intestinal Capsule: Taiwan
FJ748545	224814632	A. gobiceps isolate DL_JPN_WK4A1	worm: Japan, Wakayama
FJ748546	224814633	A. papernai isolate DL_RSA_107a	worm: South Africa
FJ748547	224814634	A. crassus isolate DL_TPE_340	worm: Taiwan
FJ748548	224814635	A. novaezelandiae isolate DL_AUS_S22	worm: Australia, Tasmania
FJ748549	224814636	A. australiensis isolate DL_TAS_60	worm: Australia, Brisbane

### Experimental infections (see also tables 4 and 5)

**Table 4:** Mean and standard deviation for parameters from infection experiments.

**Infection group**	**infection parameter**	**mean**	**sd**
50	L3	8.83	3.25
100	L3	7.67	2.58
300	L3	8.67	2.50
50	L4	14.67	3.08
100	L4	18.50	5.17
300	L4	43.33	10.63
50	Adults	3.17	1.17
100	Adults	3.67	1.75
300	Adults	12.33	2.66
50	sb. caps.	12.83	2.32
100	sb. caps.	17.33	5.28
300	sb. caps.	16.00	3.35
50	int. caps.	0.00	0.00
100	int. caps.	0.00	0.00
300	int. caps.	61.50	13.69

Infection group indicates double repeated infections with 50, 100 and 300 larvae. Sample size n = 6 for all groups. sd = standard deviation.

**Table 5:** Results of comparisons (exact Wilcoxon rank sum test) for parameters from infection experiments.

**Comparison group**	**infection parameter**	**p**	**W**
300:50	L3	0.8636	16.5
300:100	L3	0.5346	22.5
100:50	L3	0.5108	13.5
300:50	L4	0.0022	36
300:100	L4	0.0022	36
100:50	L4	0.3333	24.5
300:50	Adults	0.0022	36
300:100	Adults	0.0022	36
100:50	Adults	0.5866	22
300:50	sb. caps.	0.119	28
300:100	sb. caps.	0.619	14.5
100:50	sb. caps.	0.1645	27
300:50	int. caps.	0.0022	36
300:100	int. caps.	0.0022	36
100:50	int. caps.	1	18

Sample size n = 6 for all groups. W is the smallest sum of ranks, p indicates a significance estimate based on a two-tailed hypothesis.

No dead larvae could be found either in the intestine or in the stomach wall of the first and second group of eels, double infected with 50 or 100 L3 larvae, respectively. In the third group of eels (6 males, weight: 203 g ± 51.57, length: 52.33 cm ± 2.79; mean ± SD) which were infected twice with 300 L3 larvae after initial immunization, encapsulations were noticed not only in intestinal and swimbladder walls but also in the stomach wall. No differences in the number of living L3 larvae and encapsulated larvae in the swimbladder wall were found between the groups. Surprisingly the number of L4 larvae and of adult *A. crassus *was significantly higher in the third group double infected with 300 larvae compared to both other groups.

## Discussion

Our results for the infection of Japanese eels from SW Taiwan are in good agreement with previous findings of Münderle et al. [16] on eels from the same area: the only swimbladder nematode found was *Anguillicoloides crassus*. *Anguillicola globiceps *was not found in hosts from this area. Furthermore infection parameters with adult *A. crassus *for both wild eels and eels from aquaculture are in agreement with these previous findings. In contrast the larval prevalence as well as the larval mean abundance and larval mean intensity reported here for wild eels are surprisingly low compared to the Münderle et al. data, a fact that could possibly accord to seasonal fluctuations in the prevalence of *A. crassus *(also noticed by Münderle et al.).

We were able to show, that the infective pressure of *A. crassus *was extremely high in eels from aquaculture. This fact was indicated by slightly elevated levels of infections with adults but more importantly by strongly elevated numbers of larvae found in the swimbladder wall of cultured eels compared to wild hosts. Characteristically for macro-parasite infections [17], the frequency distribution of adult and larval *A. crassus *approaches negative binomial distribution with high degree of over-dispersion both in the wild and in cultured eels.

Regarding the higher infection parameters in aquaculture some inferences can be drawn to general aspects of host-parasite interaction in Japanese eels. Obviously, there is a sufficient number of intermediate hosts available for the larval development in aquaculture. The higher infective pressure therefore seems to be a by-product of the elevated host density. It would also seem possible, that moderately elevated levels of infection with adults increase the infective pressure on hosts, because of a relationship between adult infection and L2 larvae produced in the next generation. This second hypothesis is not supported by our data, showing a comparable percentage of hosts in which L2 larvae are present and therefore reproduction of parasites is successful in wild and cultured eels at a comparable level. This indicates a mechanism preventing *A. crassus *from fast and elevated reproduction in Japanese eels.

A high mortality of *A. crassus *larvae has been reported before in the swimbladder wall of Japanese eels [16]. We were able to show for the first time systematically, that this mortality is even more pronounced in the intestinal wall. This suggests a regulation of *A. crassus *populations by the immune-system of their natural host in east Asia (concomitant immunity), while *A. crassus *populations seem to be much more regulated by intraspecific processes in European eels [15,18]. This raises the question of how immunocompetent Japanese eels respond to infection under high infective pressure. Our data on encapsulated larvae allows some hypotheses to be formulated on the mechanisms and succession of destruction of larvae. We could clearly show that the establishment of encapsulated larvae inside the intestinal wall is related to killing of larvae in the swimbladder wall: significant numbers of encapsulated larvae in the intestinal wall were not observed when capsules in the swimbladder-wall were absent. Furthermore, no capsules in the intestinal wall could be found in single, non-repeated experimental infections of Japanese eels, while larvae are killed in the swimbladder wall. This observation shows that larvae are first encapsulated in the swimbladder wall and encapsulation inside the intestinal wall follows only repeated heavy infections. These features suggest a major role of acquired or infection induced immunity in the formation of capsules.

In the antibody response against *A. crassus*, differences in European and Japanese eels have been found. There is an earlier onset of antibody production in Japanese eels, possibly directed against larvae [19,20]. Induction of resistance was shown in immunization experiments using irradiated L3 larvae in the Japanese eel but not in the European congeners [21].

Regarding the cellular immune response against *A. crassus*, epithelioid macrophages and granulocytes have been found aggregating around larvae of European eels and forming parasitic nodules in the walls of swimbladder and intestine [22]. In these studies capsules in the swimbladder wall were more common than in the intestinal wall. For European eels conclusions from this observations, especially whether these cells are initiating the formation of connective tissue capsules [22] or just absorbing damaged tissue [23] remain debatable. Encapsulated "objects" have been noted from the intestinal wall of Japanese eels in studies comparing the immune response of Japanese and European eels [14,24].

It is advised to take into account the stomach- and intestinal wall as a second location of direct host-parasite interaction in infection experiments. In experimental studies, where L3 larvae are expelled from the copepod intermediate hosts and eels are infected through a stomach tube, capsules should be expected in the stomach wall. This seems to be an artificial situation, as the wild larvae ingested inside their copepod intermediate host are found in the intestinal wall, especially in the more posterior region. The high numbers of *A. crassus *L4 larvae and adults alive in the swimbladder of the Japanese eels experimentally double-infected with high doses of larvae demand further investigation. Possibly it is a result of the concentrated application during infection, compared to a situation in the wild or in aquaculture, where although under high infection pressure the host is gradually exposed to the larvae.

Studies on free living hosts should consider the intestinal wall as a location where dead larvae can be found. Our PCR based approach allows an identification of degraded larvae inside the swimbladder wall. This is desirable as in European eels the absence of swimbladder-capsules in the wild is assumed (mainly because these capsules are absent in experimental infections of European eels) leaving some sparsely found "objects" in the swimbladder-wall unidentified [24].

In the present study we established that it is possible to use a molecular approach to identify *A. crassus*, even if encapsulated and degraded within intestinal tissue of its eel host. We suggest a simple PCR based method providing a reliable alternative to morphological identification of *A. crassus*. We show that if swimbladder nematodes inside capsules containing degraded tissue are investigated, a PCR approach with subsequent analysis on agarose gels yields sufficient diagnostics, as the PCR product of the nematode LSU-gene can be distinguished from the chimeric amplification product of the the eel-host's LSU on an agarose gel. Subsequent sequencing of the amplified marker gene furthermore allows reliable identification of the exact parasite species, even in the absences of adult worms. To guarantee correct amplification of the nematode LSU, the collagen sheath of the capsule must be disrupted in order to allocate the nematode DNA. The inability to amplify the LSU fragment from some of the smallest capsules, may result from the already advanced degradation of the nematode DNA making it impossible to amplify long target fragments. This represents a obvious limitation of our method when applied to the most disintegrated specimen. A further development of comparable approaches using shorter target fragments for amplification can therefore be advised for authors specially interested in these issues. Nevertheless our method provides a means for identification of encapsulated objects superior to visual inspection.

## Conclusion

Based on our data we conclude that the Japanese eel is capable of killing *A. crassus *L3 larvae in the intestinal wall under high infective pressure. The observed capsules are in fact larvae killed by the first barrier of the eel immune system, which becomes effective in the case of repeated heavy infections. The extent of this phenomenon is is making it necessary to include the intestinal wall as a location of direct host-parasite interaction in future studies, also in European eels, where infective pressure is high. We therefore provide a reliable PCR-based method for species specific identification of larvae encapsulated and morphologically disintegrated by the host's immune system.

## Methods

### Sampling of eels

Cultured eels were acquired from an aquaculture directly adjacent to Kaoping river (22.6418N; 120.4440E) 15 km stream upwards from it's estuary, on the 29th of April 2008. On the same day wild eels were picked up at Tunkang Biotechnology Research Centre Fisheries Research institute in Tunkang, Pintung, Taiwan, where they had been sheltered since the time of purchase during the 2nd two weeks of April 2008 from a fisherman, fishing in the estuary of Kao-Ping river (22.5074N; 120.4220E). All eels were transported to the Institute of Fisheries Science at the National Taiwan University in Taipei in aerated plastic bags, where they were sheltered until dissection.

### Dissection of eels

Dissection of eels was carried out during May 2008. Eels were decapitated, length (to the nearest 1.0 mm) and weight (to the nearest 0.1 g) were measured, and sex was determined by visual inspection of the gonads. Stage was determined by examination of fin coloration, eye diameter and gonad development. The swimbladder was opened, adult worms were removed from the lumen with a forceps, their sex was determined, and they were counted. The wall of the swimbladder was checked for larvae using squash preparation between two Perspex plates and transmitted light under a stereo microscope. Larvae of a body length <1.5 mm were counted as L3, bigger larvae as L4. Encapsulated larvae in the swimbladder wall were counted regardless of the degree of their disintegration from the second day dissection onwards. The intestine of the eels was spread on a Perspex plate and examined for encapsulated objects using a stereo microscope and transmitted light from the third day of dissections onwards. Small parts of the intestine were intensely spread using the flat top of a bended forceps to increase translucence and visible capsules were counted. This procedure was repeated following the complete length of the intestine. A subset of the adult worms and parts of intestinal wall were fixed in DESS [25] (saturated salt solution containing DMSO) for subsequent DNA extraction.

### Analysis of infection data

Prevalence, mean abundance and mean intensity were calculated as described by Bush et al. [26] with minor modifications to assess larval parameters in more detail: Mean abundance was calculated separately as the number of larvae, number of adults and total number of worms per host. Mean intensity was calculated as number of worms (larvae, adult and total) over infected hosts (infected according to the particular parameter). Statistical analysis was carried out in R [27]. Analysis of variance was used to test differences between groups for normally distributed data (eel length and weight). Chi-square tests were employed for a direct comparison of numbers of infected individuals. Wilcoxon rank sum tests with continuity correction were used to test for significance in differences between all other non-normally distributed infection parameters. Goodness of fit tests were used to test for negative binomial distribution. Overdispersion was tested by referring the ratio of observed and theoretical variance to a Chi-square distribution. Partial correlation was used for first assessment of association between variables. For regression analysis both response and explanatory variables were log transformed to reduce heteroscedascity and to account for non-normal errors. Appropriate generalized linear models were fitted by stepwise simplification starting from maximal models.

### Amplification and sequence analysis

Individual capsules were extracted using sterile dissecting instruments (dissection pins) and transferred individually into petri dishes with deionized water. In 25 reactions the collagen sheath forming the the capsules was mechanically destroyed and the debris was transferred into 20 *μ*l microLYSIS-PLUS (Microzone Limited, Haywards Heath, UK) lysis buffer and incubated following the manufacturer's protocol. The same was done with 5 capsules not mechanically opened and as a negative control with 10 pieces of intestinal tissue showing no traces of infection. Lysates were used directly for amplification of an approximately 800 bp target fragment of the large subunit ribosomal RNA gene (LSU) by polymerase chain reaction (PCR). This PCR was performed in a reaction volume of 25 *μ*l with 0.2 *μ*M of dNTPs, 10 *μ*M of each of the primers D2A (5'-ACAAGTACCGTGAGGGAAAGT-3') and D3B (5'-TGCGAAGGAACCAGCTACTA-3') [28], 2.7 *μ*l 10×-reaction buffer, 0.1 *μ*l Taq Polymerase (Quiagen, Hilden, Germany) and 2 *μ*l sample-lysate. The thermal cycling program consisted of a denaturation at 95°C for 3 min, followed by 35 cycles of denaturation at 94°C for 30 s, annealing at 55°C for 45 s and elongation at 72°C 2 min. Followed by an additional elongation step at 72°C for 10 min. PCR amplified products were inspected on a 1.5% agarose gel. Visible bands were cut out and purified using the QiaQuick PCR purification kit (Qiagen, Hilden, Germany). 14 bands of 800 bp and 2 bands of 300 bp were sequenced in both directions using PCR primers in a concentration of 3.2 *μ*M and the BidDye terminator kit. DNA sequencing was performed on ABI 3730 DNA Analyser (Applied Biosystems, Foster City, California, USA) following the manufacturer's protocols.

Reference sequences were generated from adult *Anguillicoloides *using the same amplification protocol but direct sequencing of PCR products after a shrimp alkaline phosphatase (SAP) Exonuclease I (ExoI) clean-up. Sequencing chromatographs were post processed with trace2seq (A. Anthony and M. Blaxter, unpublished; ), consensus sequences of the forward and reverse sequences were build using the software package Mesquite [29] and deposited into GenBank database under accession numbers shown in table 3.

### Experimental infections

Uninfected Japanese eels used for the experimental infections were caught in the glass-eel stage in Taiwan by a professional fisher and transported in aerated bags to University of Karlsruhe by air mail. They were fed up in a fish lab until they reached the yellow-eel stage. The experiment was carried out between 24.05.2008 and 17.02.2009.

In total 18 Japanese eels were repeatedly infected with L3 larvae, cultivated following a procedure described in literature [30]. The eels were infected with a stomach tube containing the larvae in culture medium (RPMI 1640). Three groups of Japanese eels (N = 6) were first immunized with 50 L3 larvae. After a period of 4 months double-repeated infections in 10 days intervals with 50 (first group), 100 (second group) or 300 L3 larvae (third group) were carried out. The eels were then sheltered in 160 l tanks at 22°C before they were finally dissected 30 days after the third infection, following the procedure described above for dissection of naturally infected eels.

## Competing interests

The authors declare that they have no competing interests.

## Authors' contributions

EGH dissected the eels, noticed the unusual finding of capsules, took pictures, carried out the statistical analysis and wrote the manuscript. DRL amplified and sequenced DNA. UW performed infection experiments. HT provided close supervision throughout and contribution to the manuscript. YSH organized the acquisition of eels and supervised their dissection.
